# Emotion dysregulation in bipolar disorder according to predominant polarity

**DOI:** 10.1192/j.eurpsy.2025.1068

**Published:** 2025-08-26

**Authors:** V. Oliva, M. De Prisco, M. Bort, C. Sommerhoff, T. Fernández-Plaza, L. Montejo, V. Ruiz, M. Y. Rivas, E. Vieta, A. Murru, G. Fico

**Affiliations:** 1 Department of Medicine, Faculty of Medicine and Health Sciences, University of Barcelona (UB); 2 Bipolar and Depressive Disorders Unit, Institute of Neuroscience, Hospital Clinic, IDIBAPS, CIBERSAM, University of Barcelona (UB), Barcelona, Spain

## Abstract

**Introduction:**

Emotion dysregulation (ED) is a core feature of bipolar disorder (BD), impacting patients’ ability to manage intense emotional responses and maintain mood stability. Predominant polarity (PP) is a clinical specifier in BD with important implications for prognosis, treatment planning, and outcomes. PP categorizes patients based on the mood episode type they experience most frequently: depressive PP (DPP), manic PP (MPP), or undetermined PP (UPP) when no episode type prevails. Given that PP reflects the types of symptoms most commonly experienced by patients, and that ED is closely linked to mood symptoms in BD, it remains unclear whether ED differs across PP subgroups.

**Objectives:**

We aimed to compare overall levels of ED and specific emotion regulation (ER) domains across patients with BD and DPP, MPP, and UPP.

**Methods:**

We analyzed data from patients with BD enrolled in the Bipolar Exposome-Gene Interaction Naturalistic (BEGIN) study. ED and ER strategy alterations were assessed using the Difficulties in Emotion Regulation Scale (DERS-28) and its subscales: Lack of Control, Non-Acceptance, Interference, Inattention, and Confusion. PP was defined based on the Barcelona proposal. Due to non-normal DERS score distributions (Shapiro-Wilk p < 0.05), the Kruskal-Wallis test was applied to compare DERS total and subscale scores across PP groups, followed by Dunn’s test with Bonferroni correction for pairwise comparisons.

**Results:**

The sample included 64 patients with BD (mean age=51±12.5; 46.9% female). Of these, 17 had DPP, 7 had MPP, and 40 had UPP. Significant differences across PP groups were observed in overall ED (p=0.04), with the UPP group showing higher total DERS scores than the MPP group (Z=2.55, p=0.03). In the Lack of Control subscale, both the DPP and UPP groups scored higher than the MPP group (Z=2.43, p=0.04 and Z=3.14, p=0.01, respectively). No other significant differences were found.

**Conclusions:**

These findings indicate that ED varies across PP groups, emphasizing the potential advantages of tailored interventions within a precision psychiatry framework. Personalized approaches to managing ED may improve long-term outcomes and decrease the risk of recurrence across mood states in BD.

**Disclosure of Interest:**

None Declared

